# Discovery of new 3-methylquinoxalines as potential anti-cancer agents and apoptosis inducers targeting VEGFR-2: design, synthesis, and *in silico* studies

**DOI:** 10.1080/14756366.2021.1945591

**Published:** 2021-07-29

**Authors:** Mohammed M. Alanazi, Elwan Alaa, Nawaf A. Alsaif, Ahmad J. Obaidullah, Hamad M. Alkahtani, Abdulrahman A. Al-Mehizia, Sultan M. Alsubaie, Mohammed S. Taghour, Ibrahim H. Eissa

**Affiliations:** a Department of Pharmaceutical Chemistry, College of Pharmacy, King Saud University, Riyadh, Saudi Arabia; b Pharmaceutical Medicinal Chemistry and Drug Design Department, Faculty of Pharmacy (Boys), Al-Azhar University, Cairo, Egypt

**Keywords:** Anticancer, apoptosis, in silico studies, 3-methylquinoxalin-2(1H)-one, 3-methylquinoxaline-2-thiol, VEGFR-2 inhibitors

## Abstract

There is an urgent need to design new anticancer agents that can prevent cancer cell proliferation even with minimal side effects. Accordingly, two new series of 3-methylquinoxalin-2(1*H*)-one and 3-methylquinoxaline-2-thiol derivatives were designed to act as VEGFR-2 inhibitors. The designed derivatives were synthesised and evaluated *in vitro* as cytotoxic agents against two human cancer cell lines namely, HepG-2 and MCF-7. Also, the synthesised derivatives were assessed for their VEGFR-2inhibitory effect. The most promising member **11e** were further investigated to reach a valuable insight about its apoptotic effect through cell cycle and apoptosis analyses. Moreover, deep investigations were carried out for compound **11e** using western-plot analyses to detect its effect against some apoptotic and apoptotic parameters including caspase-9, caspase-3, BAX, and Bcl-2. Many *in silico* investigations including docking, ADMET, toxicity studies were performed to predict binding affinity, pharmacokinetic, drug likeness, and toxicity of the synthesised compounds. The results revealed that compounds **11e, 11g, 12e, 12g,** and **12k** exhibited promising cytotoxic activities (IC_50_ range is 2.1 − 9.8 µM), comparing to sorafenib (IC_50_ = 3.4 and 2.2 µM against MCF-7 and HepG2, respectively). Moreover, **11b, 11f, 11g, 12e, 12f, 12g,** and **12k** showed the highest VEGFR-2 inhibitory activities (IC_50_ range is 2.9 − 5.4 µM), comparing to sorafenib (IC_50_ = 3.07 nM). Additionally, compound **11e** had good potential to arrest the HepG2 cell growth at G2/M phase and to induce apoptosis by 49.14% compared to the control cells (9.71%). As well, such compound showed a significant increase in the level of caspase-3 (2.34-fold), caspase-9 (2.34-fold), and BAX (3.14-fold), and a significant decrease in Bcl-2 level (3.13-fold). For *in silico* studies, the synthesised compounds showed binding mode similar to that of the reference compound (sorafenib).

## 1. Introduction

Cancer has been the most difficult and life-threatening illness to be treated^1^. After cardiovascular disease (CVD) cancer has been reported to be a significant cause of death worldwide^2^. At the end of 2018, cancer caused 9.6 million deaths^3^. The current anticancer therapy has many side effects arising from non-selectivity of the development of drug resistance^4^. Nonetheless, there is an urgent need to design new anticancer drugs that can prevent cancer cell proliferation even with minimal to no side effects on healthy cells.

At the level of molecular biology, protein tyrosine kinases have an important role in cell proliferation, migration, survival, and progression^5^. Tyrosine kinases phosphorylate the protein's tyrosine residues resulting in altered protein function. In some cases, tyrosine kinases become continuously active which ultimately leads to cancer^6^. Tyrosine kinase receptors (RTKs) are a panel of cell surface receptors that transfer the signal to polypeptides, hormones, and growth factors^7^. There have been numerous known RTKs such as Vascular endothelial growth factor receptors (VEGFRs) and endothelial growth factor receptors (EGFRs)^8^.

VEGFRs have been recognised as an outstanding medicinal target to discover new anticancer agents^9^^,^^10^. The class of VEGFRs comprises three subtypes; VEGFR-1, VEGFR-2, and VEGFR-3^11^. Among them, VEGFR-2 which has a crucial role in tumour angiogenesis. VEGFR-2 can be activated through binding with VEGF which starts the process of phosphorylation which boosts proliferation and migration of the endothelial cells^12^. VEGFR-2 is mainly overexpressed throughout endothelial cells of the tumour vasculature, with less expression in normal endothelial cells^13^. Hepatocellular carcinoma and breast cancer are well-known examples of tumours with overexpressed VEGFR-2^14–16^.

VEGFR-2 inhibitors are small molecules that bind at the ATP binding site of VEGFR-2 to inhibit angiogenesis and lymphangiogenesis^17^. Beside many VEGFR-2 inhibitors approved by FDA or under clinical trials, there are a lot of effort to discover new ones for the management of cancer. Sorafenib **I** is a bi-aryl urea, has inhibitory effect against tyrosine kinases involved in tumour development, including VEGFR-2^18^. Sunitinib **II** is anti-tumour drug with dual activity against VEGFR-2 and PDGFR-β^19^. Telatinib **III** is an orally active anti-VEGFR-2 small-molecule^20^. Nintedanib **IV** is a potent small-molecule tyrosine kinase inhibitor with oral activity. In addition, it has a triple angiokinase inhibitory effect as it inhibits the three major signalling pathways involved in angiogenesis^21^. Acrizanib **V** is a VEGFR-2 inhibitor with limited systemic exposure after topical ocular administration^22^. Vorolanib **VI** is a novel indolinone-based kinase inhibitor that targets the VEGFR-2^23^. It has fewer adverse effects and a wide therapeutic window^24^.

**Figure 1.**
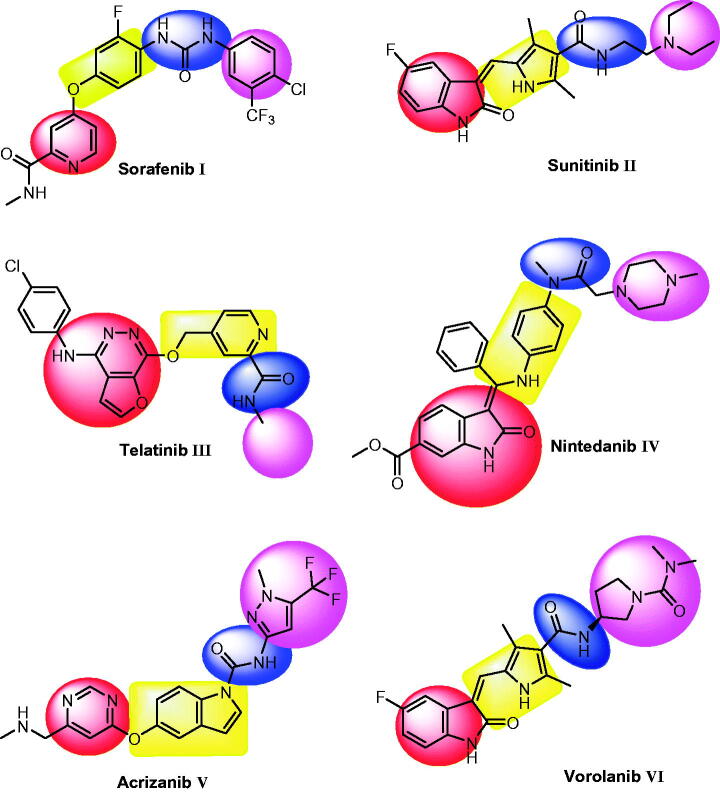
Some reported VEGFR-2 inhibitors and their basic pharmacophoric features.

**Figure 2.**
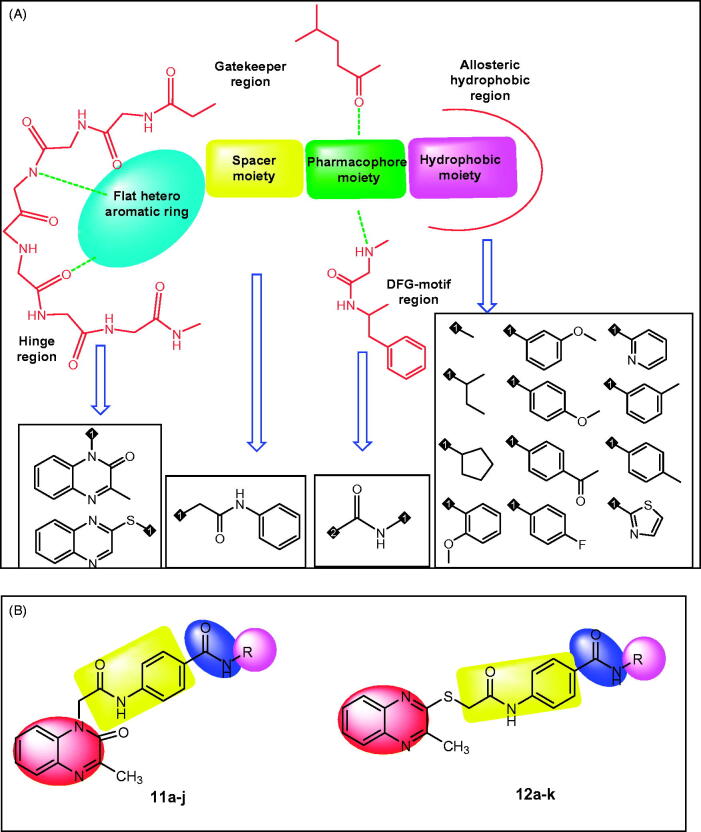
A) Different bio-isosteres that can occupy the ATP binding site of VEGFR-2. B) Representative examples of the new synthesised compounds having the same essential pharmacophoric features of VEGFR-2 inhibitor.

Essential Pharmacophoric features of VEGFR-2 inhibitors have been reported in many publications^25–30^. The reported pharmacophore includes: i) a flat hetero aromatic moiety which binds Cys917 via a hydrogen bonding interaction^26^, **(ii)** a spacer moiety which occupies the area between the ATP-binding domain and the DFG domain^31^, **(iii)** a pharmacophore moiety which consists of H-bond acceptor (HBA) and one H-bond donor (HBD) groups (e.g. amide or urea). Both HBA and HBD have a key binding role, as they form hydrogen bonding interactions with two crucial residues (Glu883 and Asp1044) ^32^, and **(iv)** a terminal hydrophobic moiety can make many hydrophobic interactions in the allosteric hydrophobic pocket of VEGFR-2^33^ (Figures 1 and 2).

In the current work, ligand-based drug design approach^34–37^ was used to synthesise two series of 3-methylquinoxalin-2(1*H*)-one and 3-methylquinoxaline-2-thiol derivatives. This work is an extension of the earlier activities of our team to synthesise effective anticancer agents targeting VEGFR-2^9^^,^^38^^,^^39^. The synthesised derivatives were evaluated *in vitro* and *in silico* to assess their VEGFR-2 inhibitory activities.

### 1.1. Rationale of molecular design

VEGFR-2 inhibitors competitively block the ATP binding site which consists of four main regions. i) Hinge region which is occupied by the flat hetero aromatic ring of VEGFR-2 inhibitors. ii) Gatekeeper region which is occupied by the spacer moiety of VEGFR-2 inhibitors. iii) DFG-motif region which is occupied by the pharmacophore moiety of VEGFR-2 inhibitors. iv) Allosteric hydrophobic region which is occupied by the terminal hydrophobic moiety of VEGFR-2 inhibitors^33^^,^^40–42^ (Figure 2).

The main objective of our design was the synthesis of new compounds having the main pharmacophoric features of VEGFR-2 inhibitors. Such compounds comprise different bio-isosteres, each of them occupy a specific region at ATP binding site.

For the hinge region, two quinoxaline moieties were used; i) 3-methylquinoxalin-2(1*H*)-one (compounds **11a-j**) and ii) 3-methylquinoxaline-2-thiol (compounds **12a-k**). The bicyclic structure of quinoxaline moiety is suitable to the large size space of the ATP binding region^43^. In addition, the nitrogen atoms act as hydrogen-bond acceptors to facilitate the hydrogen bonding interaction with the hinge region. The Gatekeeper region was targeted to be occupied by *N*-phenylacetamide moiety as spacer group. Regarding the DFG-motif region, an amide group (pharmacophore moiety) was selected to be buried in it. Finally, the allosteric hydrophobic region can be occupied by different aliphatic and aromatic derivatives to study the structure-activity relationships (Figure 2).

## 2. Results and discussion

### 2.1. Chemistry

**Scheme 1.**
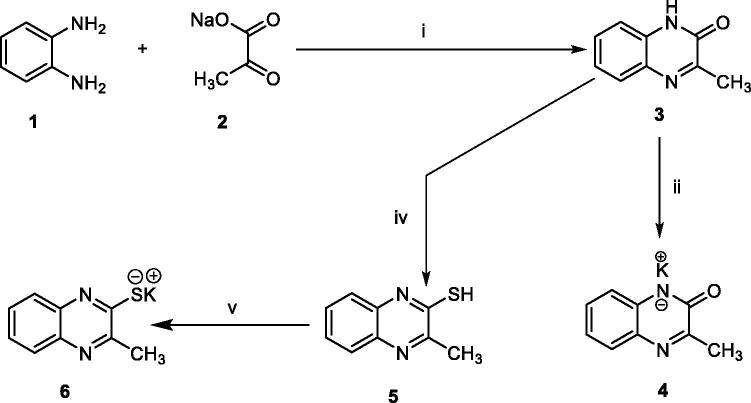
synthesis of compound key potassium salts **4** and **5. Reagents and conditions**: i) g. acetic acid/H_2_O/reflux/2 h, ii) Alc. KOH/reflux/30 min., iii) i) P_2_S_5_/pyridine/reflux/2 h then HCl, v) Alc. KOH/reflux/30 min.

**Scheme 2.**
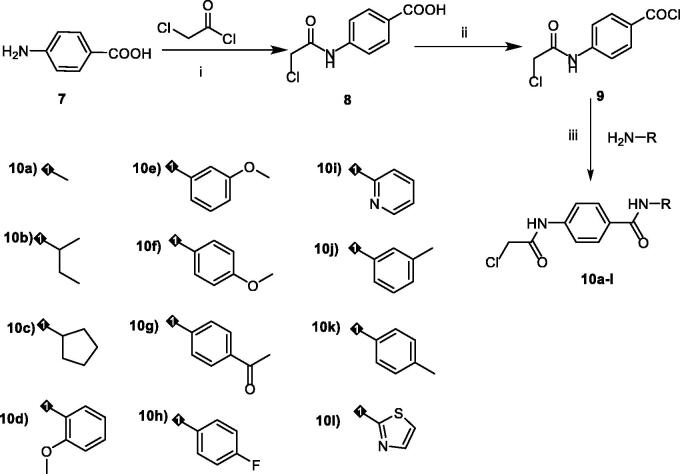
synthesis of the key intermediates **10a-l. Reagents and conditions:** i) DMF/NaHCO_3_/stirring, r.t./1h, ii) dichloroethane/SOCl_2_/DMF/reflux/1h, iii) CH3CN/stirring/r.t./3 h.

**Scheme 3.**
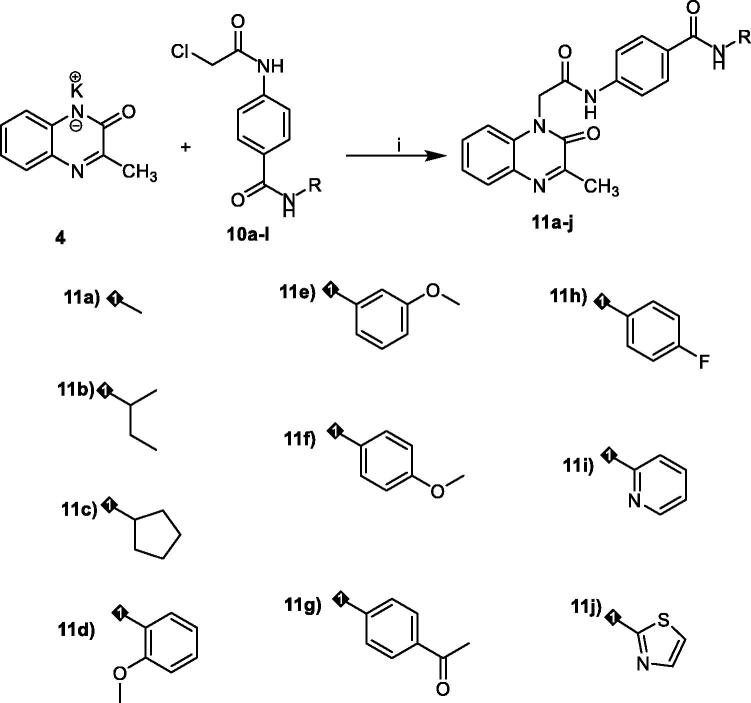
synthesis of the target compounds **11a-j. Reagents and conditions:** i) KI/DMF/heating/W.B./8h.

**Scheme 4.**
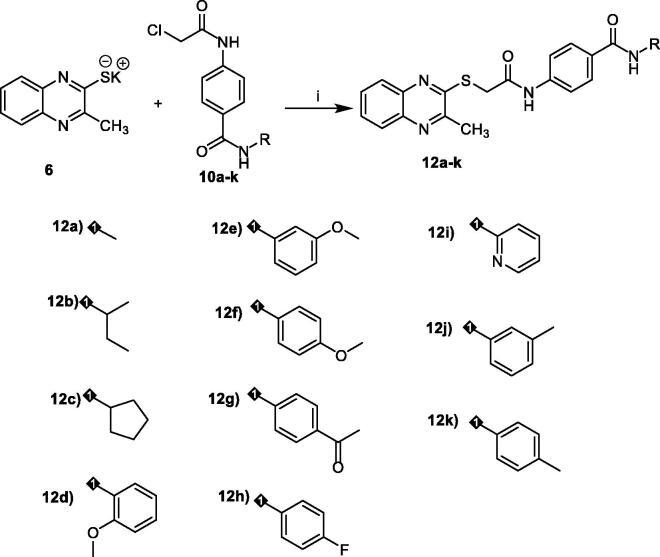
synthesis of the target compounds **12a-k. Reagents and conditions:** i) KI/DMF/heating/W.B./8h.

In order to synthesise the designed compounds, Schemes 1–4 were adopted. Initially, *o*-phenylenediamine **1** was refluxed with sodium pyruvate **2** to afford 3-methylquinoxalin-2(1*H*)-one **3** according to the reported procedure^44^. Subsequent heating of compound **3** with alcoholic potassium hydroxide gave the corresponding potassium salt **4**^44^. To prepare 3-methylquinoxaline-2-thiol **5**, the previously prepared 3-methylquinoxalin-2(1*H*)-one **3** was refluxed with P_2_S_5_ in pyridine as a solvent, then the reaction was acidified using HCl^45^^,^^46^. Heating of compound **5** with alcoholic potassium hydroxide gave the corresponding potassium salt **6** (Scheme 1).

The key intermediates were synthesised as described in Scheme 2. The commercially available *p*-amino benzoic acid **7** was treated with chloroacetyl chloride in dry DMF in the presence of NaHCO_3_ to afford 4–(2-chloroacetamido)benzoic acid **8**. Chlorination of **8** was achieved by its reflux with SOCl_2_ in dichloroethane in the presence of catalytic amount of dry DMF to give the key compound 4–(2-chloroacetamido)benzoyl chloride **9**. At the end, in acetonitrile and TEA mixture, compound **9** was stirred at room temperature with appropriate amines namely, methylamine, sec-butylamine, cyclopentylamine, 2-methoxyaniline, 3-methoxyaniline, 4-methoxyaniline, 4-aminoacetophenone, 4-fluoroaniline, 2-aminopyridine, *m*-toluidine, *p*-toluidine, and 2-aminothiazole to give the corresponding key intermediates **10a-l**, respectively. The IR spectra of the key intermediates **10a-l** exhibited the appearance of absorption bands at the ranges of 3254 − 3326 cm^−1^ and 1702 − 1625 cm^−1^ due to the NH and 2C=O groups, respectively. In addition, ^1^H NMR analyses exhibited the appearance of characteristic singlet signals for amidic NHs around *δ* 10.00 ppm. Also, it showed singlet signals for CH_2_ protons of acetamide moiety around *δ* 4.30 ppm. Besides, such CH_2_ group was detected around *δ* 44.02 ppm in ^13 ^C NMR spectra.

The first series of the target compounds was prepared as described in Scheme 3. The potassium salt of 3-methylquinoxalin-2(1*H*)-one **4** was heated in dry DMF with the keys intermediates in the presence of catalytic amount of KI to give the titled compounds **11a-j**.

The second series of the target compounds was synthesised depending on the synthetic pathway described in Scheme 4. The potassium salt of methylquinoxaline-2-thiol **6** was heated in dry DMF with the keys intermediates **10a-k** in the presence of catalytic amount of KI to give the titled compounds **12a-k**.

^1^H NMR spectra exhibited the presence of singlet signals of CH_3_ group of 3-methylquinoxalin-2(1*H*)-one moiety around δ 2.49 ppm. In addition, ^1^H NMR spectra showed characteristic signals for additional aromatic and aliphatic protons at the expected chemical shift. Taken compound **11c** and **12c** as representative examples, it showed many characteristic signals at aliphatic region corresponding to cyclopentyl moiety. Moreover, ^13 ^C NMR spectra of such compounds confirmed the previous results as the aliphatic protons of cyclopentyl moiety appeared at the aliphatic region.

### 2.2. Biological testing

#### 2.2.1. *In vitro* cytotoxic activities

Cytotoxic activities of the synthesised compounds were evaluated against MCF-7 (human breast cancer cell line) and HepG2 (human liver carcinoma cell line) using MTT assay^47^, using sorafenib as a reference standard (Table 1). Among the target compounds, **11e, 11g, 12e, 12g,** and **12k** exhibited promising cytotoxicity against the two cell lines with IC_50_ values ranging from 2.1 to 9.8 µM, comparing to sorafenib (IC_50_ = 3.4 and 2.2 µM against MCF-7 and HepG2, respectively). Compound **11e** exhibited a superior activity against MCF-7 and HepG2 with IC_50_ values of 2.7 and 2.1, respectively. In addition, compounds **11f** and **12f** showed promising activity against HepG2 cells with IC_50_ values of 9.6 and 7.5, respectively. Compounds **11a, 11b, 11c, 12c,** and **12d** showed moderate activity against HepG2 cells with IC_50_ values of 16.5, 12.8, 18.7, 11.4, 18.7, and 17.6 µM, respectively. In addition, compounds **11f** and **12f** showed moderate activity against MCF-7 cells with IC_50_ values of 12.4 and 10.8 µM, respectively. On the other hand, compounds **11d, 11h, 11i, 11j, 12a,** and **12b** showed weak cytotoxic activity against the two cell lines.

**Table 1.** *In vitro* cytotoxic and VEGFR-2inhibitory activities.

Comp.	Cytotoxicity IC_50_ ( µM )^a^		VEGFR-2 inhibitory activity IC_50_ ( nM )^a^
	MCF-7	HepG2	
**11a**	23.9	16.5	11.2
**11b**	21.2	12.8	5.3
**11c**	28.1	18.7	12.7
**11d**	52.3	34.1	37.4
**11e**	2.7	2.1	2.6
**11f**	12.4	9.6	4.8
**11g**	6.7	3.8	3.4
**11h**	25.8	22.7	11.6
**11i**	69.7	51.8	52.7
**11j**	32.8	27.8	13.4
**12a**	69.2	23.7	32.7
**12b**	35.7	21.7	15.7
**12c**	31.3	18.7	19.8
**12d**	23.5	17.6	13.4
**12e**	5.3	4.8	3.8
**12f**	10.8	7.5	3.8
**12g**	8.7	6.1	5.4
**12h**	67.8	35.8	51.4
**12i**	58.6	44.7	24.1
**12j**	61.2	40.7	37.8
**12k**	9.8	6.7	2.9
**Sorafenib**	3.4	2.2	3.1

^a^All IC_50_ values are calculated as the mean of at least three different experiments.

#### 2.2.2. Vegfr-2 inhibitory assay

VEGFR-2 inhibitory activity of the synthesised compounds was investigated using sorafenib as a reference drug. Table 1. Summarised the IC_50_ values of VEGFR-2 growth inhibitory concentration for all the synthesised members.

Compound **11e** and **12k** exhibited VEGFR-2 inhibitory activity (IC_50_ = 2.6 and 2.9 nM, respectively) higher than that of sorafenib (IC_50_ = 3.07 nM). Additionally, compounds **11b, 11f, 11g, 12e, 12f, 12g,** and **12k** showed promising activities with IC_50_ values ranging from 2.9 to 5.4 nM. On the other hand, compounds **11a, 11c, 11d, 11h, 11i, 11j, 12a, 12b, 12c, 12d, 12h, 12i,** and **12j** showed moderate to weak activity. Its IC_50_ values are ranging from 11.2 to 52.7 nM.

#### 2.2.3. Statistical correlation between VEGFR-2 inhibition and cytotoxicity

**Figure 3.**
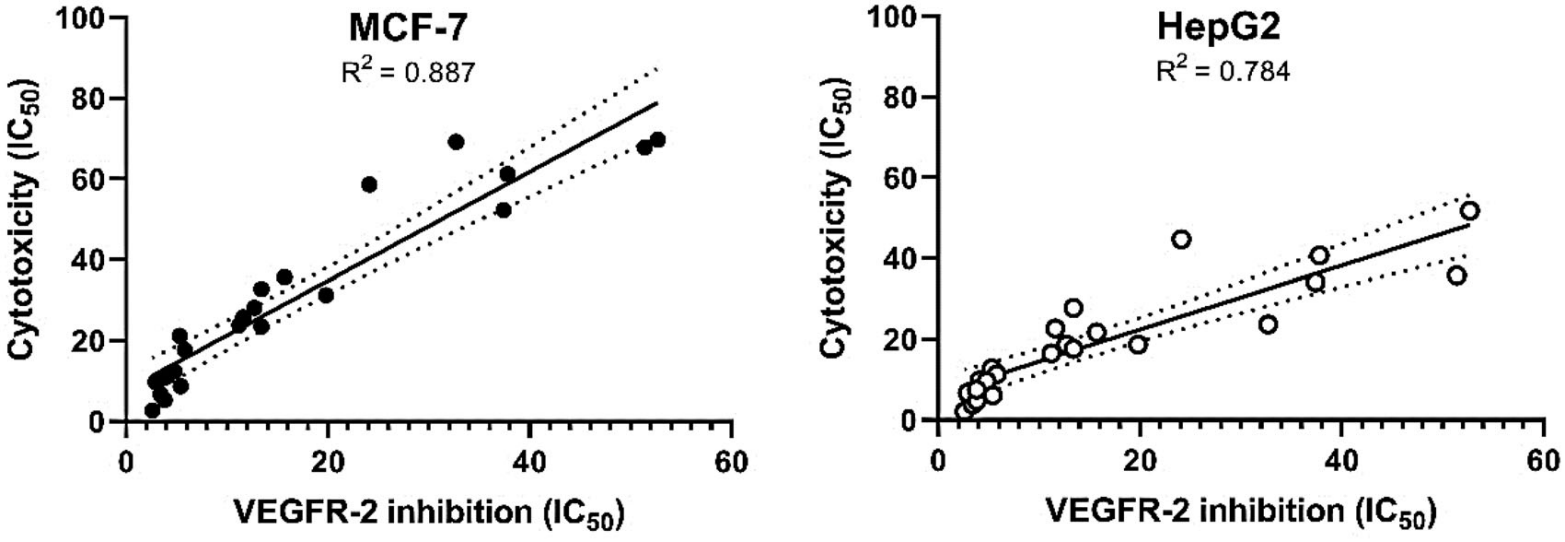
Simple linear regression for the correlation between VEGFR-2 inhibition and cytotoxicity.

To study the relation between cytotoxicity and VEGFR-2 inhibition, we plotted the values of VEGFR-2 inhibition against the corresponding cytotoxicity results using simple linear regression analysis. Co-efficient of determination (R^2^) were calculated in this analysis. It was found that R^2^ of VEGFR-2 inhibition and MCF-7 cytotoxicity is 0.887 with *p* values >0.0001. In addition, R^2^ of VEGFR-2 inhibition and HepG2 cytotoxicity is 0.887 with *p* values >0.0001. The results indicated that there are high correlations between VEGFR-2 inhibition and cytotoxic activity on both cell lines, which reveals that the cytotoxicity may be a result of VEGFR-2 inhibition (Figure 3).

#### 2.2.4. Structure-Activity relationship (SAR)

The results of different biological analyses (cytotoxic activity and VEGFR-2 inhibitory assay) gave a valuable SAR. Initially, the effect of the flat hetero aromatic ring on the activity was explored. Comparing the cytotoxic activity of compounds **11a-j** (incorporating 3-methylquinoxalin-2(1*H*)-one) with compounds **12a-k** (incorporating 3-methylquinoxaline-2-thiol**)** indicated that 3-methylquinoxalin-2(1*H*)-one moiety is more advantageous than 3-methylquinoxaline-2-thiol moiety for cytotoxic and VEGFR-2 inhibitory effects.

Then, we investigated the effect of the terminal hydrophobic moiety. Comparing the activity of compounds **11a-c** and **12a-c** having aliphatic hydrophobic moieties with compounds **11e-g** and **12e-g** having aromatic hydrophobic moieties indicated that aromatic moieties are beneficial for activity. For aliphatic moieties, there is no great variation in the activity among small size (compound **11a** and **12a**), bulk (compound **11c** and **12c**), and branched (compound **11b** and **12b**) aliphatic moieties.

In addition, the effect of the substitution on the aromatic hydrophobic moieties has been investigated. Comparing the activity of compound **12k** (incorporating 4-methylphenyl moiety) and compounds **11f** and **12f** (incorporating 4-methoxyphenyl moiety) with compounds **11h** and **12h** (incorporating 4-fluorophenyl) and compounds **11g** and **12g** (incorporating acetophenone moiety), revealed that grafting an electron donating group is more preferred biologically than electron withdrawing one. For electron withdrawing groups, it was found that acetyl incorporating members (**11g** and **12g**) are more active than fluoro incorporating one (**11h** and **12h**).

Next, the effect of the substitution on the aromatic hydrophobic moieties with electron donating group has been examined. For methylquinoxalin-2(1*H*)-one derivatives, the activities reduced in the order of 3-methoxy (**11e**) > 4-methoxy (**11f**) > 2-methoxy (**11d**). With regard to 3-methylquinoxaline-2-thiol derivatives, the activities decreased in the order of 4-methyl (**12k**) > 3-methoxy (**12e**) > 4-methoxy (**12f**) > 2-methoxy (**12d**) > 3-methoxy (**12j**).

Finally, the decreased IC_50_ values of compound **11j** (with thiazole moiety) against the tested cell lines and VEGFR-2 than the IC_50_ values of compound **11i (**with pyridine moiety**),** indicated that five-membered hetero aromatic hydrophobic moiety is more efficient than six- membered one.

#### 2.2.5. In vitro *cytotoxicity against normal cell*

The cytotoxicities of the most active compounds (**11e** and **12e)** against primary rat hepatocytes (normal hepatic cells) were evaluated *in vitro* (Table 2). The results revealed that the tested compounds have low toxicity against the tested cells with IC_50_ values of 15.0 and 16.7 μM, respectively. Sorafenib as a reference drug showed IC_50_ value of 13.4 µM against the tested cells. These results revealed that the synthesised compounds have low toxicity against the normal cells as their toxicities are comparable to that of FDA approved drug (sorafenib).

**Table 2.** *In vitro* cytotoxicity of the most active compounds (**11e** and **12e**) and sorafenib against normal cells (primary rat hepatocytes).

Comp.	IC_50_ (µM)^a^ primary rat hepatocytes
**11e**	15.0
**12e**	16.7
**Sorafenib**	13.4

^a^IC_50_ values are the mean of three separate experiments.

#### 2.2.6. Cell cycle analysis

The cell cycle is a well-maintained process by which cells of eukaryotes replicate themselves. The homeostatic balance between cell loss and cell gain must be achieved to produce and conserve the complex architecture of tissues. One way in which this connection may be achieved is through the coupling of the cell cycle and apoptosis^48^.

**Figure 4.**
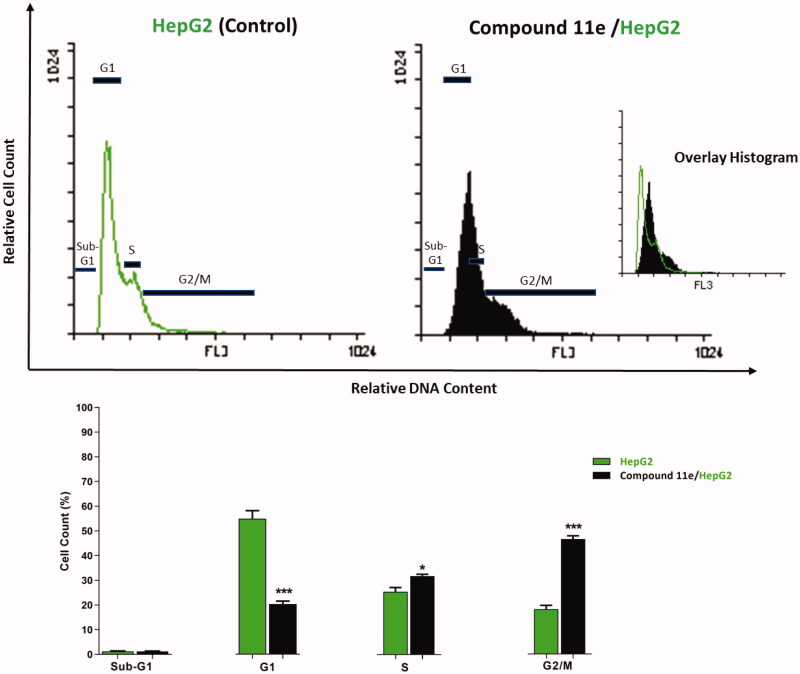
Flow cytometric analysis of cell cycle phases post the compound **11e** treatment.

Since compound **11e** effectively inhibited the growth of HepG2 cells, it was expected that this inhibitory effect was due to its capability to hinder the cell cycle progression. Therefore, cell cycle process was analysed after exposure of HepG2 cells to **11e** with a concentration of 2.1 µM (IC_50_ value of compound **11e**) after 24 h. HepG2 cells were used as a control without treatment by compound **11e.** Flow cytometry data revealed that the percentage of cells arrested at the G2/M phase increased from 18.24% (in control cells) to 46.62 (in **11e** treated cells). In addition, the percentage of HepG2 cells mild increased at the S phase from 25.48 to 31.80%. Oppositely, the percentage of HepG2 cells decreased at G1 phase from 55.03% to 20.34%. Such findings revealed that compound **11e** arrested the HepG2 cell growth at G2/M phase (Figure 4 and Supplementary Data).

#### 2.2.7. Apoptosis analysis

To quantify the apoptosis triggered by **11e**, Annexin-V/propidium iodide (PI) staining assay was conducted. In such procedure, compound **11e** at a concentration of 2.1 µM was applied on HepG2 cells. Then, the media were incubated for 24 h. As shown in Table 3, the apoptotic effect of **11e** in HepG2 cells was five times more than observed in control cells. In details, compound **11e** induced apoptosis by 49.14% (early apoptosis = 48.87% & late apoptosis = 0.27%), compared to 9.71% in the control cells (early apoptosis = 9.58% & late apoptosis = 0.13%).

**Table 3.** Effect of compound **11e** on stages of the cell death process in HepG2 cells after 24 h treatment.

Sample	Viable^a^ (Left Bottom)	Apoptosis^a^		Necrosis^a^ (Left Top)
		Early (Right Bottom)	Late (Right Top)	
HepG2	90.20 ± 1.07	9.58 ± 0.86	0.13 ± 0.01	0.10 ± 0.02
Compound 11e /HepG2	50.65 ± 3.66	48.87 ± 3.69	0.27 ± 0.04	0.20 ± 0.03

^a^Values are reported as mean ± SD of three different experiments.

#### 2.2.8. Western blot analysis

Apoptosis is a programmed cell death characterised by some biological processes including condensation of nuclear chromatin, loss of plasma membrane phospholipid asymmetry, DNA cleavage into small fragments, and formation of membrane-bound apoptotic bodies^49^.

During intrinsic apoptosis, caspase-9 is activated to produce a subsequent activation of other effector caspases. It was reported that caspase-9 is a highly specific protease that only cleaves a few proteins, whereas caspase-3 contributes to the majority of cleavage that takes place during apoptosis^50^^,^^51^. Additionally, the mitochondrial apoptosis is largely mediated through Bcl-2 family proteins, which include. i) Pro-apoptotic members such as BAX that promote mitochondrial permeability and cell death. ii) Anti-apoptotic members such as Bcl-2 that inhibit the mitochondrial release of cyt *c* and suppress cell death^52^. According to these reports, a cell with a high BAX/Bcl-2 ratio will be more sensitive to some given apoptotic stimuli when compared to a similar cell type with a low BAX/Bcl-2ratio^53^.

##### 2.2.8.1. Caspase-3 and caspase-9 determination

**Figure 5.**
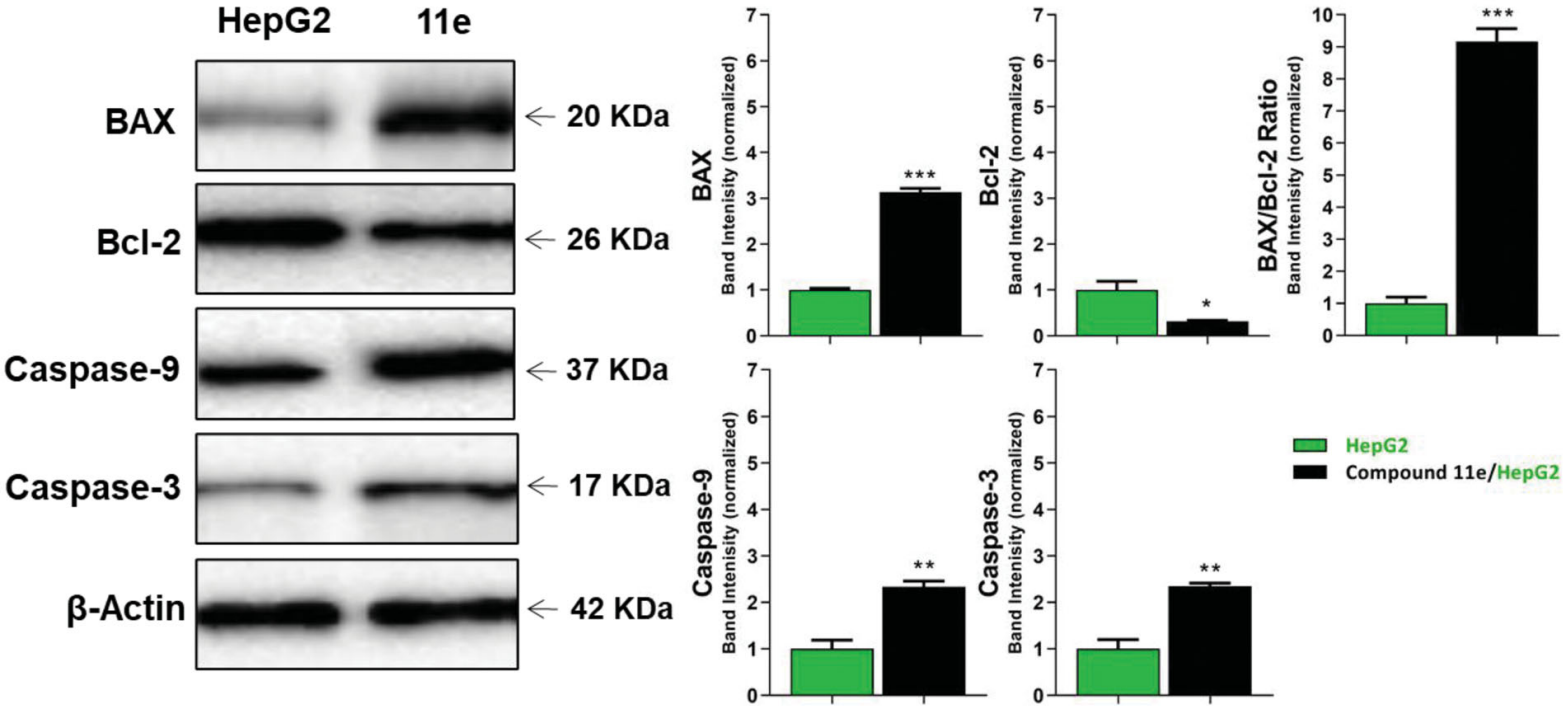
The immunoblotting of BAX, Bcl-2, Caspase-9, and Caspase-3 (Normalized to β-actin).

To investigate the effect of the synthesised compounds on caspase-3 and caspase-9 levels, the most promising member **11e** was applied on the most sensitive cells (HepG2) at a concentration of 2.1 µM for 24 h. Western blot analyses revealed that compound **11e** produced a significant increase in the level of caspase-3 (2.34-fold) compared to the control cells. Moreover, compound **11e** showed a significant increase in the level of caspase-9 (2.34-fold) compared to the control cells (Figure 5 and Supplementary Data).

##### 2.2.8.2. BAX and Bcl-2 determination

Compound **11e** as a promising member was investigated to evaluate its effect on BAX and Bcl-2 after 24 h of its application on HepG2 cells using Western blot technique. The results showed that compound **11e** produced an up-regulation of BAX and down-regulation of Bcl-2. In details, BAX level increased by 3.14-fold, while Bcl-2 level decreased by 3.13-fold. In addition, BAX/Bcl-2 ratio was 9.17, which indicated that compound **11e** had a significant effect on apoptosis pathway (Figure 5 and Supplementary Data).

### 2.3. *In silico* studies

#### 2.3.1. Docking studies

In this work, the synthesised compounds were docked against VEGFR-2 using sorafenib as a reference drug. This investigational work was performed to get further insight into the binding modes of the synthesised compounds against VEGFR-2 binding site (PDB ID: 2OH4). The binding free energies (**ΔG)** for all the synthesised compounds against the target receptor were calculated and reported in Table 4. The reported key binding site of VEGFR-2 consists of Glu883 and Asp1044^33^^,^^54^. Validation of the docking procedure and the binding mode of the reference drug (sorafenib)^33^^,^^54^ were showed in Supplementary data.

**Table 4.** Binding free energies (ΔG in Kcal/mol) of the synthesised compounds and sorafenib against VEGFR-2

Comp. No.	ΔG [Kcal/mol]	Compound	ΔG [Kcal/mol]
**11a**	−22.37	**12b**	−27.03
**11b**	−28.35	**12c**	−28.20
**11c**	−28.03	**12d**	−28.95
**11d**	−27.90	**12e**	−27.98
**11e**	−28.81	**12f**	−29.16
**11f**	−28.79	**12g**	−30.11
**11g**	−30.62	**12h**	−28.26
**11h**	−27.26	**12i**	−27.58
**11i**	−27.00	**12j**	−29.46
**11j**	−25.38	**12k**	−28.73
**12a**	−21.81	**Sorafenib**	−25.69

**Figure 6.**
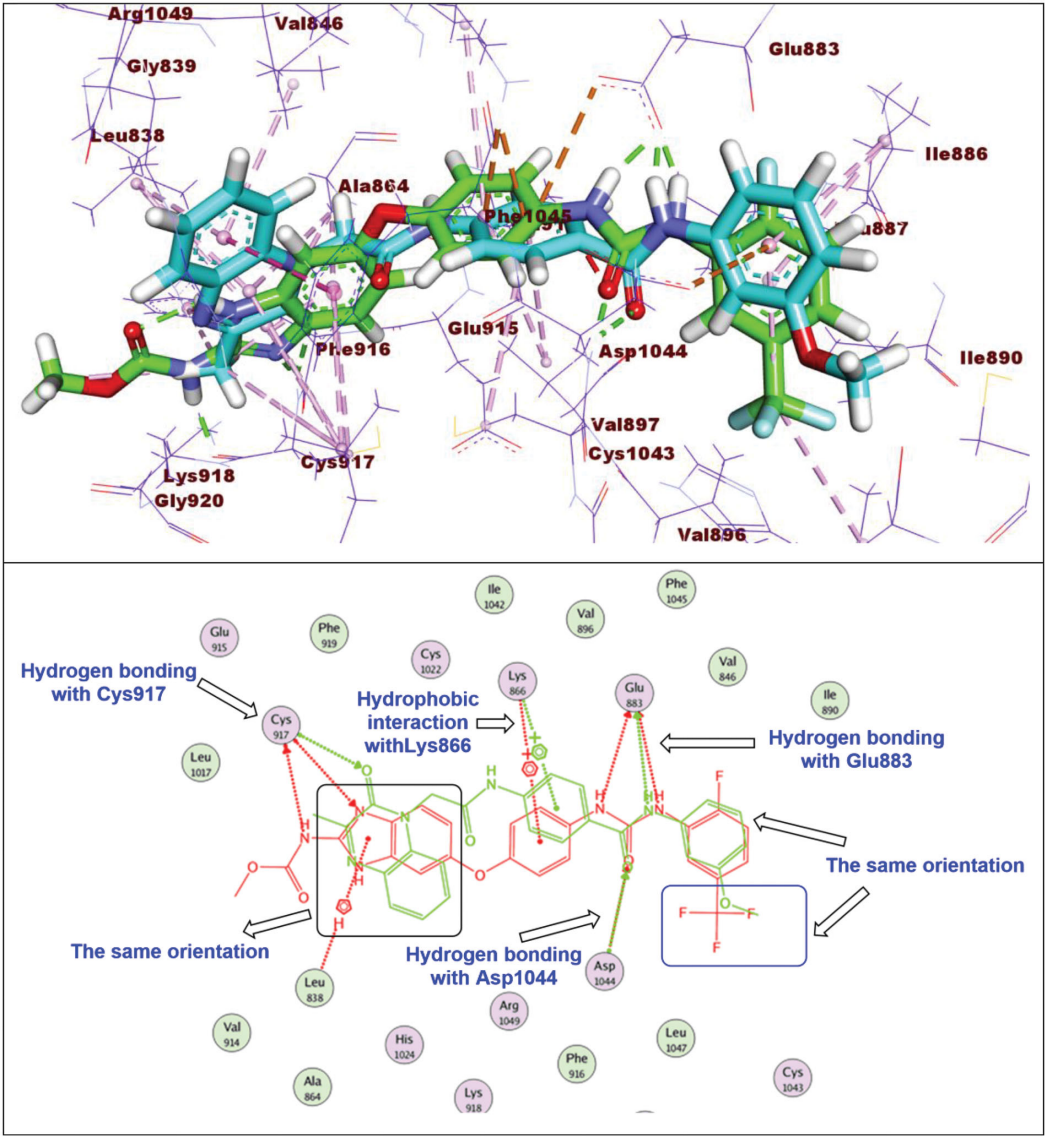
Superimposition of compound **11e** and sorafenib inside the active sites of VEGFR-2. Compound **11e** was completely buried inside VEGFR-2 active site with similar binding mode to sorafenib.

The synthesised compounds exhibited binding mode inside the binding sites of VEGFR-2 similar to that of sorafenib. Compound **11e** was completely buried inside VEGFR-2 active site with similar binding mode to sorafenib. The docking score of such compound was −28.81 kcal/mol. The pharmacophore moiety (amide group) was incorporated in hydrogen bonding interaction forming a hydrogen bond with Glu883 and another one with Asp1044. The phenyl group (spacer) formed three hydrophobic interactions with Val914 and Cys1043. The 3-methylquinoxalin-2(1*H*)-one nucleus was inserted in hinge region of the binding pocket forming five hydrophobic interactions with Leu1033, Phe916, Ala864, and Leu838. In addition, the terminal methoxyphenyl group (hydrophobic tail) formed one hydrophobic bond with Leu887. Additionally, it formed two electrostatic interactions with Asp1044 (Figure 6).

**Figure 7.**
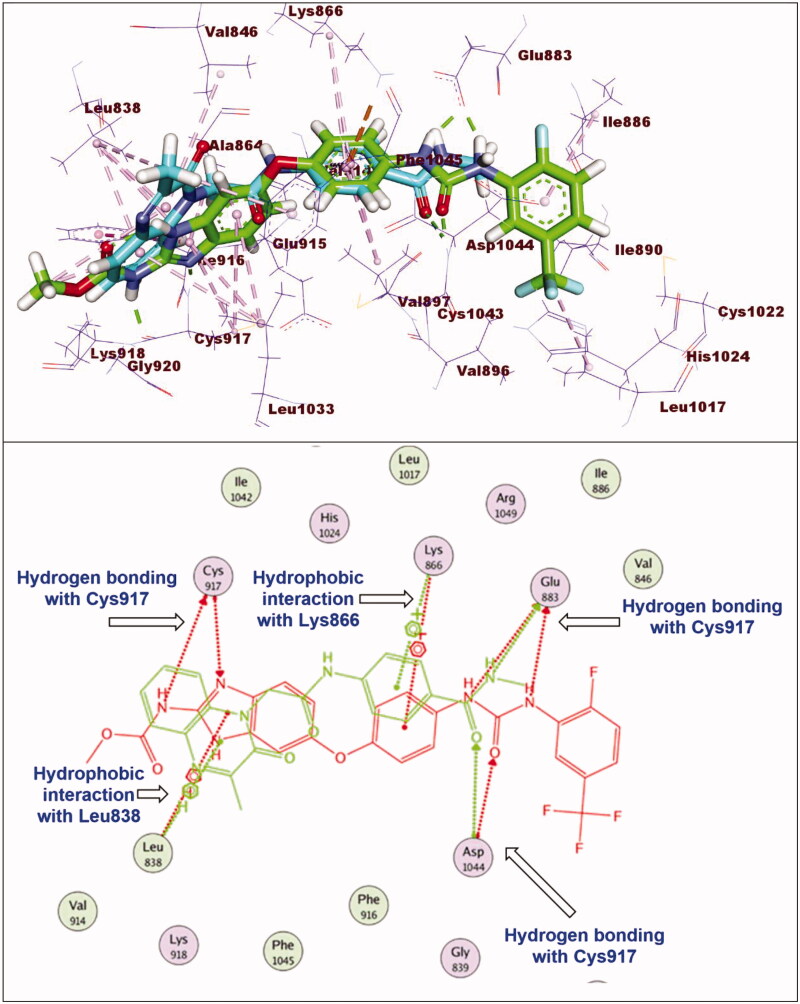
Superimposition of compound **11a** and sorafenib inside the active sites of VEGFR-2. Compound **11a** showed binding mode similar to that of sorafenib with lack of hydrophobic interaction inside the allosteric binding pocket and absence of hydrogen bonding interaction with Cys917 at hinge region.

Regarding compound **11a** (incorporating methyl group as hydrophobic tail) showed binding energy of −22.37 kcal/mol. It showed binding mode similar to that of sorafenib with some deviations. Firstly, due to lack of bulk aromatic moiety (as appeared in compound **11e**), this led to disappearance of hydrophobic interactions at the allosteric binding pocket of VEGFR-2. In addition, the orientation of 3-methylquinoxalin-2(1*H*)-one nucleus at the hinge region prevent the hydrogen bonding interaction with Cys917 (Figure 7).

**Figure 8.**
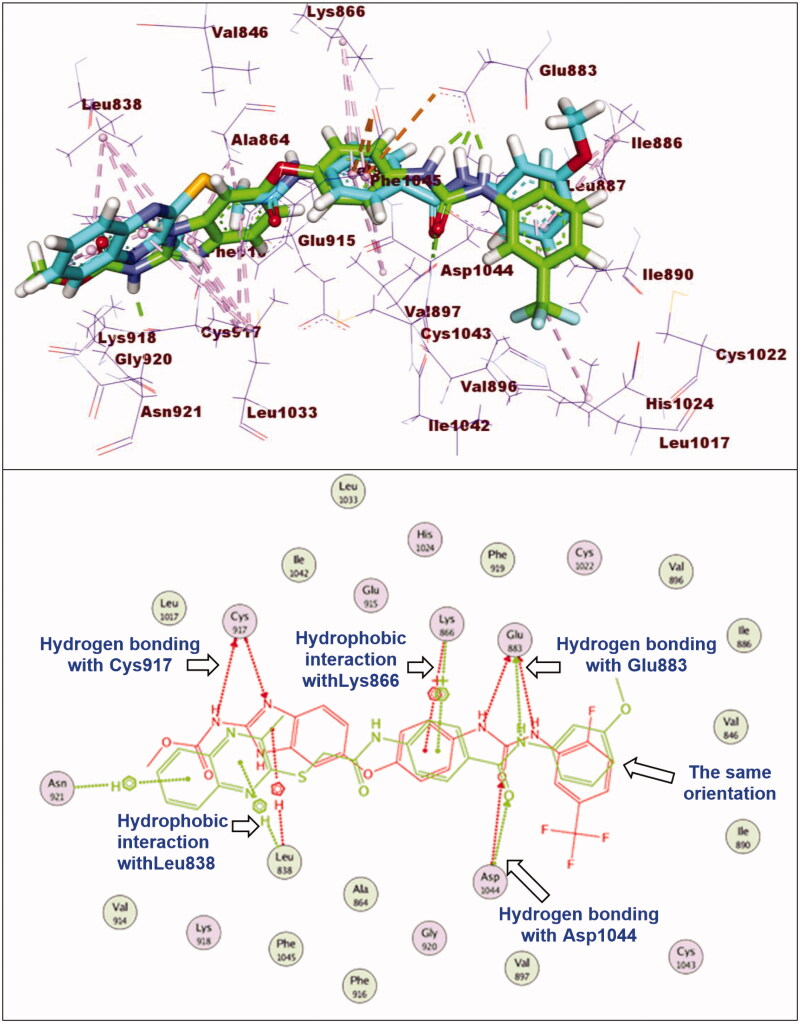
Superimposition of compound **12e** and sorafenib inside the active sites of VEGFR-2. Compound **12e** showed binding mode similar to that of sorafenib with lack of hydrogen bonding interaction with Cys917 at hinge region.

With respect to compound **12a**, it exhibited a binding mode like that of sorafenib with binding energy of −27.98 kcal/mol. The different features of compounds **12a** occupied the same regions which occupied by sorafenib. However, elongation of the linker moiety exerted mild change in the orientation of 3-methylquinoxaline-2-thiol nucleus at the hinge region preventing the hydrogen bonding interaction with Cys917 (Figure 8).

#### 2.3.2. *In silico* ADMET studies

The *in silico* ADMET parameters were assessed via Discovery studio 4.0 using Sorafenib as a reference molecule.

**Figure 9.**
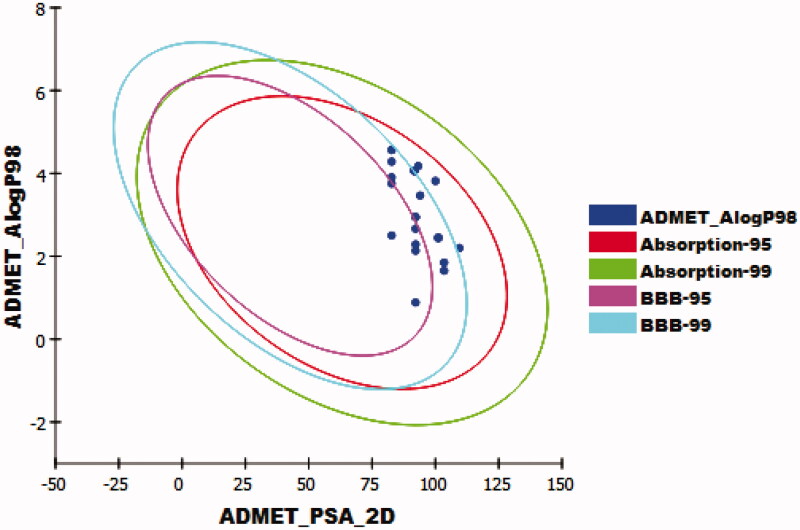
The expected ADMET study.

The results revealed that the tested compounds have low or very low BBB penetration levels except for compounds **12h, 12j,** and **12k** which were predicted to have medium levels. Accordingly, most compounds were anticipated to be safe against CNS. Furthermore, compounds **11a-g, 11i,** and **11j** were predicted to have good levels of aqueous solubility, while compounds **12a-k** were predicted to have low levels. Moreover, intestinal absorption levels of all the tested compounds were predicted to be good. The cytochrome P4502D6 inhibition was predicted using CYP2D6 model^55^. All the tested compounds were predicted as non-inhibitors of CYP2D6. So that, these compounds are expected to be safe for the liver. The plasma protein binding (PPB) model predicts the degree of molecule binding to PP. If it is > = 90%, it means that a molecule can bind the PP at high concentration^56^. Compounds **11c-e, 11g-i, 12h, 12i,** and **12k** were expected to bind plasma protein over 90%, while compounds **11a, 11b, 11f, 11j, 12a-g,** and **12i** were predicted to bind plasma protein less than 90% (Table 5, Figure 9).

**Table 5.** Calculated ADMET descriptors

Comp.	BBB level^a^	Solubility level^b^	Absorption level^c^	CYP2D6 prediction^d^	PPB prediction^e^
**11a**	3	3	0	X	X
**11b**	3	3	0	X	X
**11c**	3	3	0	X	√
**11d**	3	3	0	X	√
**11e**	3	3	0	X	√
**11f**	3	3	0	X	X
**11g**	4	3	0	X	√
**11h**	3	2	0	X	√
**11i**	3	3	0	X	√
**11j**	3	3	0	X	X
**12a**	3	2	0	X	X
**12b**	2	2	0	X	X
**12c**	2	2	0	X	X
**12d**	4	2	0	X	X
**12e**	4	2	0	X	X
**12f**	4	2	0	X	X
**12g**	4	2	0	X	X
**12h**	2	2	0	X	√
**12i**	3	2	0	X	X
**12j**	2	2	0	X	√
**12k**	2	2	0	X	√
**Sorafenib**	4	1	0	X	√

^a^BBB means blood brain barrier which may be very high (0), high (1), medium (2), low (3), or very low (4).

^b^Solubility level may be very low (1), low (2), good (3), or optimal (4).

^c^Absorption level may be good (0), moderate (1), poor (2), or very poor (3).

^d^CYP2D6 means cytochrome P2D6 which may be inhibitor (√) or non-inhibitor (X).

^e^PBB means plasma protein binding which may be less than 90% (X) or more than 90% (√).

#### 2.3.3. *In silico* toxicity studies

Discovery studio 4.0 was used to determine the expected toxicity potential of the synthesised compounds^57^^,^^58^.

As shown in Table 6, most compounds showed *in silico* low toxicity profile against the tested models. Compounds **11a-j** and **12a,b** were predicted to have carcinogenic potency TD_50_ values ranging from 19.427 to 81.588 mg/kg body weight/day, which were higher than that of sorafenib (carcinogenic potency TD_50_ = 19.236 mg/kg body weight/day). While compounds **12c-k** were estimated to have low carcinogenic potency TD_50_ values (from 7.026 to 17.638 mg/kg body weight/day). In addition, the rat maximum tolerated doses of compounds **11h, 12b,** and **12h-k** were estimated to be between 0.133 and 0.096 g/kg body weight, which were higher than that of sorafenib (rat maximum tolerated dose = 0.089 g/kg body weight). The other derivatives were predicted to have fewer rat maximum tolerated doses. Moreover, compounds **11a-c, 11g, 11i, 11j, 12a, 12c, 12g, 12i,** and **12k** were predicted to be non-toxic against developmental toxicity potential model. For rat oral LD_50_ model, the tested compounds showed oral LD_50_ values ranging from 2.102 to 18.807 g/kg body weight/day. Such values are far more than that of sorafenib (oral LD_50_ = 0.823 g/kg body weight/day). Moreover, all the tested compounds were predicted to be mild irritant against ocular irritancy model, and non-irritant against skin irritancy model.

**Table 6.** Toxicity properties of the synthesised compounds.

Comp.	Carcinogenic Potency TD_50_ (Mouse)^a^	Rat Maximum Tolerated Dose (Feed)^b^	Developmental Toxicity Potential	Rat Oral LD_50_^b^	Ocular Irritancy	Skin Irritancy
**11a**	81.588	0.056	Non-Toxic	6.255	Mild	Non-Irritant
**11b**	68.438	0.076	Non-Toxic	15.151	Mild	Non-Irritant
**11c**	33.606	0.063	Non-Toxic	7.417	Mild	Non-Irritant
**11d**	48.211	0.043	Toxic	4.609	Mild	Non-Irritant
**11e**	62.412	0.043	Toxic	9.772	Mild	Non-Irritant
**11f**	37.877	0.043	Toxic	6.249	Mild	Non-Irritant
**11g**	51.779	0.060	Non-Toxic	8.214	Mild	Non-Irritant
**11h**	26.216	0.103	Toxic	6.088	Mild	Non-Irritant
**11i**	32.099	0.070	Non-Toxic	5.862	Mild	Non-Irritant
**11j**	40.916	0.048	Non-Toxic	9.349	Mild	Non-Irritant
**12a**	23.269	0.072	Non-Toxic	6.323	Mild	Non-Irritant
**12b**	19.427	0.104	Toxic	8.109	Mild	Non-Irritant
**12c**	9.528	0.080	Non-Toxic	4.965	Mild	Non-Irritant
**12d**	13.624	0.056	Toxic	2.102	Mild	Non-Irritant
**12e**	17.638	0.056	Toxic	4.761	Mild	Non-Irritant
**12f**	10.704	0.056	Toxic	3.045	Mild	Non-Irritant
**12g**	14.619	0.081	Non-Toxic	4.779	Mild	Non-Irritant
**12h**	7.416	0.133	Toxic	4.066	Mild	Non-Irritant
**12i**	9.093	0.100	Non-Toxic	3.499	Mild	Non-Irritant
**12j**	11.577	0.096	Toxic	11.265	Mild	Non-Irritant
**12k**	7.026	0.096	Non-Toxic	15.912	Mild	Non-Irritant
**Sorafenib**	19.236	0.089	Toxic	0.823	Mild	Non-Irritant

^a^Unit: mg/kg body weight/day.

^b^Unit: g/kg body weight.

## 3. Conclusion

Twenty-two compounds were designed, synthesised, and evaluated as VEGFR-2 inhibitors. Such derivatives were assessed against MCF-7 and HepG-2 cell lines to estimate its antiproliferative activities. Compounds **11e, 11g, 12e, 12g,** and **12k** displayed promising cytotoxic activities against MCF-7 and HepG-2 with IC_50_ values ranging from 2.1 to 9.8 µM. Furthermore, compounds **11b, 11e, 11f, 11g, 12e, 12f, 12g,** and **12k** showed VEGFR-2 inhibitory activities with IC_50_ values of 5.3, 2.6, 4.8, 3.4, 3.8, 3.8, 5.4, and 2.9 nM, respectively. SAR revealed that 3-methylquinoxalin-2(1*H*)-one moiety is more beneficial than 3-methylquinoxaline-2-thiol moiety for cytotoxicity and VEGFR-2 inhibitory activities. Also, the terminal aromatic moieties were found to be more valuable than the terminal aliphatic ones. Compound **11e,** the most potent member, arrested the HepG2 cell growth at G2/M phase and induced apoptosis by 49.14% compared to the control cells (9.71%). Additionally, such derivative showed a significant elevation in the level of caspase-3 (2.34-fold) and caspase-9 (2.34-fold). Moreover, it showed a marked increase in BAX (3.14-fold) and a significant reduction in Bcl-2 level (3.13-fold). The *in silico* studies revealed that the synthesised compounds showed binding interactions like that of sorafenib with good drug likeness profile.

## 4. Experimental

### 4.1. Chemistry

#### 4.1.1. General

Reagents, solvent, and apparatus used in chemical synthesis were showed in Supplementary data. Compounds **3, 4, 5, 6, 8, 9, 10a-l** were prepared according to reported procedures^44–46^^,^^59–64^. Physical, elemental, and spectral data of the intermediate compounds **10a-l** were depicted in Supplementary data.

***General procedure for synthesis of compounds 11a-j***

To a solution of the potassium salt of 3-methylquinoxalin-2(1*H*)-one **4** (320 mg, 0.002 mol) in DMF (20 ml) the appropriate 4–(2-chloroacetamido)-*N*-substituted-benzamides **10a-i and 10l** (0.002 mol) was added. The mixture was heated on a water bath for 10 h. After cooling to room temperature, the reaction mixture was poured onto crushed ice. The precipitated solids were filtered, dried and crystalised from ethanol to give the target compounds **11a-j.**

##### 4.1.1.1. N-Methyl-4–(2-(3-methyl-2-oxoquinoxalin-1(2H)-yl)acetamido)benzamide 11a

White powder (yield 75%); mp: 294–297 °C; FT-IR (v max, cm^−1^): 3288 (NH), 1674, 1655 (C=O), 1601 (C = N); ^1^H NMR (700 MHz, DMSO-*d*_6_) δ 10.68 (s, 1H), 8.34 (q, *J* = 4.6 Hz, 1H), 7.83 − 7.79 (m, 3H), 7.64 (d, *J* = 8.8 Hz, 2H), 7.57 (t, *J* = 8.5 Hz, 1H), 7.52 (d, *J* = 8.4 Hz, 1H), 7.38 (t, *J* = 7.5 Hz, 1H), 5.16 (s, 2H), 2.76 (s, 3H), 2.49 (s, 3H); ^13 ^C NMR (176 MHz, DMSO) δ 166.46, 165.68, 157.97, 154.84, 141.46, 133.46, 132.46, 130.18, 129.87, 129.27, 128.47 (2 C), 123.92, 118.81(2 C), 115.19, 45.75, 26.66, 21.59; MS (*m/z*): 351 (M^+^ + 1, 15% %), 201 (60%); Anal. Calcd. for C_19_H_18_N_4_O_3_ (350.38): C, 65.13; H, 5.18; N, 15.99; Found: C, 65.53; H, 5.06; N, 15.64%.

##### 4.1.1.2. N-(sec-Butyl)-4–(2-(3-methyl-2-oxoquinoxalin-1(2H)-yl)acetamido)benzamide 11b

White powder (yield 70%); mp: 319–321 °C; FT-IR (v max, cm^−1^): 3275 (NH), 2965 (CH aliphatic), 1660, 1634 (C=O), 1600 (C = N); ^1^H NMR (700 MHz, DMSO-*d*_6_) δ 10.68 (s, 1H), 8.04 (d, *J* = 8.2 Hz, 1H), 7.85 − 7.82 (m, 2H), 7.81 (dd, *J* = 8.0, 1.5 Hz, 1H), 7.66 − 7.62 (m, 2H), 7.57 (ddd, *J* = 8.6, 7.1, 1.5 Hz, 1H), 7.53 (dd, *J* = 8.5, 1.3 Hz, 1H), 7.38 (ddd, *J* = 8.2, 7.1, 1.3 Hz, 1H), 5.16 (s, 2H), 3.91 (ddd, *J* = 14.1, 7.6, 6.2 Hz, 1H), 2.49 (s, 3H), 1.58 − 1.44 (m, 2H), 1.13 (d, *J* = 6.6 Hz, 3H), 0.86 (t, *J* = 7.4 Hz, 3H); ^13 ^C NMR (176 MHz, DMSO-*d*_6_) δ 165.66, 165.44, 157.96, 154.85, 141.39, 133.47, 132.46, 130.22, 130.18, 129.27, 128.65 (2 C), 123.92, 118.69 (2 C), 115.20, 46.81, 45.75, 29.32, 21.59, 20.77, 11.25; MS (*m/z*): 393 (M^+^ + 1, 80%); Anal. Calcd. for C_22_H_24_N_4_O_3_ (392.46): C, 67.33; H, 6.16; N, 14.28; Found: C, 66.94; H, 6.34; N, 13.95%.

##### 4.1.1.3. N-Cyclopentyl-4–(2-(3-methyl-2-oxoquinoxalin-1(2H)-yl)acetamido)benzamide 11c

Brown powder (yield 72%); mp: 298 − 300 °C; FT-IR (v max, cm^−1^): 3281 (NH), 2953 (CH aliphatic), 1631 (C=O), 1603 (C=N); ^1^H NMR (700 MHz, DMSO-*d*_6_) δ 10.69 (s, 1H), 8.18 (d, *J* = 7.6 Hz, 1H), 7.84 (d, *J* = 8.2 Hz, 2H), 7.80 (d, *J* = 8.0 Hz, 1H), 7.64 (d, *J* = 8.4 Hz, 2H), 7.57 (t, *J* = 7.7 Hz, 1H), 7.53 (d, *J* = 8.4 Hz, 1H), 7.37 (t, *J* = 7.5 Hz, 1H), 5.16 (s, 2H), 4.22 (h, *J* = 6.6 Hz, 1H), 2.49 (s, 3H), 1.91 − 1.84 (m, 2H), 1.69 (m, 2H), 1.53 (m, *J* = 7.4 Hz, 4H); ^13 ^C NMR (176 MHz, DMSO-*d*_6_) δ 165.70, 165.66, 157.95, 154.84, 141.41, 133.46, 132.46, 130.17, 130.09, 129.27 (2 C), 128.72, 123.90, 118.66 (2 C), 115.18, 51.35, 45.75, 32.61 (2 C), 24.09 (2 C), 21.58; MS (*m/z*): 405 (M^+^ + 1, 50% %), 330 (100%); Anal. Calcd. for C_23_H_24_N_4_O_3_ (404.47): C, 68.30; H, 5.98; N, 13.85; Found: C, 68.65; H, 6.13; N, 13.52%.

##### 4.1.1.4. N-(2-Methoxyphenyl)-4–(2-(3-methyl-2-oxoquinoxalin-1(2H)-yl)acetamido)benzamide 11d

Grey powder (yield 74%); mp: 265 − 267 °C; FT-IR (v max, cm^−1^): 3289, 3040 (NH), 2922 (CH aliphatic), 1659 (C=O), 1602 (C = N); ^1^H NMR (700 MHz, DMSO-*d*_6_) δ 10.77 (s, 1H), 9.33 (s, 1H), 7.98 − 7.93 (m, 2H), 7.80 (ddd, *J* = 19.3, 7.9, 1.6 Hz, 2H), 7.75 − 7.70 (m, 2H), 7.58 (ddd, *J* = 8.5, 7.0, 1.5 Hz, 1H), 7.54 (dd, *J* = 8.5, 1.3 Hz, 1H), 7.39 (ddd, *J* = 8.1, 7.0, 1.3 Hz, 1H), 7.18 (td, *J* = 7.8, 1.7 Hz, 1H), 7.10 (dd, *J* = 8.3, 1.4 Hz, 1H), 6.97 (td, *J* = 7.7, 1.4 Hz, 1H), 5.18 (s, 2H), 3.84 (s, 3H), 2.49 (s, 3H); ^13 ^C NMR (176 MHz, DMSO-*d*_6_) δ 165.81, 165.25, 159.87, 157.97, 154.85, 142.03, 140.91, 133.47, 132.47, 130.20, 130.01, 129.82, 129.29, 129.22 (2 C), 123.94, 118.82 (2 C), 115.21, 112.98, 109.49, 106.45, 55.46, 45.80, 21.59; MS (*m/z*): 443 (M^+^ + 40%); Anal. Calcd. for C_25_H_22_N_4_O_4_ (442.48): C, 67.86; H, 5.01; N, 12.66; Found: C, 68.03; H, 4.77; N, 12.36%.

##### 4.1.1.5. N-(3-Methoxyphenyl)-4–(2-(3-methyl-2-oxoquinoxalin-1(2H)-yl)acetamido)benzamide 11e

Grey powder (yield 70%); mp: 237 − 239 °C; FT-IR (v max, cm^−1^): 3273 (NH), 2922 (CH aliphatic), 1655 (C=O), 1604 (C = N); ^1^H NMR (700 MHz, DMSO-*d*_6_) δ 10.77 (s, 1H), 10.11 (s, 1H), 7.98 − 7.94 (m, 2H), 7.81 (dd, *J* = 8.1, 1.5 Hz, 1H), 7.72 (d, *J* = 8.7 Hz, 2H), 7.58 (td, *J* = 7.7, 6.9, 1.5 Hz, 1H), 7.54 (d, *J* = 8.4 Hz, 1H), 7.47 (t, *J* = 2.2 Hz, 1H), 7.41 − 7.35 (m, 2H), 7.25 (t, *J* = 8.1 Hz, 1H), 6.68 (dd, *J* = 8.3, 2.5 Hz, 1H), 5.18 (s, 2H), 3.76 (s, 3H), 2.49 (s, 3H); ^13 ^C NMR (176 MHz, DMSO-*d*_6_) δ 165.81, 165.25, 159.87, 157.97, 154.85, 142.03, 140.91, 133.47, 132.47, 130.20, 130.01, 129.82, 129.29, 129.22(2 C), 123.94, 118.82 (2 C), 115.21, 112.98, 109.49, 106.45, 55.46, 45.80, 21.59; MS (*m/z*): 443 (M^+^ + 1, 40% %); Anal. Calcd. for C_25_H_22_N_4_O_4_ (442.48): C, 67.86; H, 5.01; N, 12.66; Found: C, 67.50; H, 4.95; N, 12.25%.

##### 4.1.1.6. N-(4-Methoxyphenyl)-4–(2-(3-methyl-2-oxoquinoxalin-1(2H)-yl)acetamido)benzamide 11f

Yellow crystals (yield 76%); mp: 324 − 227 °C; FT-IR (v max, cm^−1^): 3269 (NH), 2957, 2834 (CH aliphatic), 1655 (C=O), 1601 (C = N); ^1^H NMR (700 MHz, DMSO-*d*_6_) δ 10.76 (s, 1H), 10.03 (s, 1H), 7.97 − 7.95 (m, 2H), 7.81 (dd, *J* = 8.0, 1.5 Hz, 1H), 7.73 − 7.70 (m, 2H), 7.68 − 7.66 (m, 2H), 7.58 (ddd, *J* = 8.5, 7.0, 1.5 Hz, 1H), 7.54 (dd, *J* = 8.5, 1.3 Hz, 1H), 7.38 (ddd, *J* = 8.1, 7.1, 1.3 Hz, 1H), 6.95 − 6.90 (m, 2H), 5.18 (s, 2H), 3.75 (s, 3H), 2.51 (s, 3H); ^13 ^C NMR (176 MHz, DMSO-*d*_6_) δ 165.77, 164.81, 157.97, 155.92, 154.85, 141.84, 133.47, 132.76, 132.47, 130.19, 130.14, 129.28 (2 C), 129.08, 123.94, 122.41(2 C), 118.82(2 C), 115.20, 114.18(2 C), 55.63, 45.79, 21.59; MS (*m/z*): 443 (M^+^ + 1, 100%); Anal. Calcd. for C_25_H_22_N_4_O_4_ (442.48): C, 67.86; H, 5.01; N, 12.66; Found: C, 67.33; H, 4.97; N, 12.22%.

##### 4.1.1.7. N-(4-Acetylphenyl)-4–(2-(3-methyl-2-oxoquinoxalin-1(2H)-yl)acetamido)benzamide 11g

White crystals (yield 71%); mp: 313 − 315 °C; FT-IR (v max, cm^−1^): 3281 (NH), 2921 (CH aliphatic), 1659 (C=O), 1603 (C = N); ^1^H NMR (700 MHz, DMSO-*d*_6_) δ 10.82 (s, 1H), 10.46 (s, 1H), 8.02 − 7.96 (m, 4H), 7.95 (d, *J* = 9.0 Hz, 2H), 7.81 (dd, *J* = 7.9, 1.5 Hz, 1H), 7.79 − 7.73 (m, 2H), 7.58 (ddd, *J* = 8.5, 6.9, 1.5 Hz, 1H), 7.54 (dd, *J* = 8.6, 1.5 Hz, 1H), 7.38 (ddd, *J* = 8.2, 7.0, 1.4 Hz, 1H), 5.19 (s, 2H), 2.55 (s, 3H), 2.49 (s, 3H); ^13 ^C NMR (176 MHz, DMSO-*d*_6_) δ 197.05, 165.87, 165.64, 157.96, 154.85, 144.19, 142.34, 133.46, 132.47, 132.35, 130.19, 129.76 (2 C), 129.57, 129.46 (2 C), 129.29, 123.93, 119.84(2 C), 118.87 (2 C), 115.20, 45.81, 26.94, 21.59; Anal. Calcd. for C_26_H_22_N_4_O_4_ (454.49): C, 68.71; H, 4.88; N, 12.33; Found: C, 69.03; H, 4.71; N, 11.92%.

##### 4.1.1.8. N-(4-Fluorophenyl)-4–(2-(3-methyl-2-oxoquinoxalin-1(2H)-yl)acetamido)benzamide 11h

White powder (yield 72%); mp: 260 − 262 °C; FT-IR (v max, cm^−1^): 3274 (NH), 3038 (CH aromatic), 2945 (CH aliphatic), 1666, 1640 (C=O), 1605 (C = N); ^1^H NMR (700 MHz, DMSO-*d*_6_) δ 10.88 (s, 1H), 10.63 (s, 1H), 8.44 (d, *J* = 8 Hz, 1H), 8.24 (d, *J* = 8 Hz, 1H), 8.03 (m, 2H), 7.83 − 7.74 (m, 2H), 7.73 (s, 1H), 7.63 (t, *J* = 10.3 Hz, 2H), 7.63 − 7.51 (m, 1H), 7.41 − 7.34 (m, 2H), 4.94 (s, 2H), 2.49 (s, 3H); ^13 ^C NMR (176 MHz, DMSO-*d*_6_) δ 165.84, 165.43, 157.96, 154.85, 151.48, 147.63, 146.74, 139.02, 138.28, 133.46, 132.46, 131.23, 131.02, 130.20, 129.28, 125.86, 123.95, 119.16, 118.88, 118.78, 116.21, 115.18, 63.52, 21.58; MS (*m/z*): 431 (M^+^ + 1, 30% %), 201 (100%); Anal. Calcd. for C_24_H_19_FN_4_O_3_ (430.44): C, 66.97; H, 4.45; N, 13.02; Found: C, 66.57; H, 4.34; N, 12.74%.

##### 4.1.1.9. 4–(2-(3-Methyl-2-oxoquinoxalin-1(2H)-yl)acetamido)-N-(pyridin-2-yl)benzamide 11i

Buff powder (yield 70%); mp: 292 − 294 °C; FT-IR (v max, cm^−1^): 3438 (NH), 1660 (C=O), 1600 (C = N); ^1^H NMR (700 MHz, DMSO-*d*_6_) δ 10.78 (s, 1H), 8.31 (s, 1H), 7.90 (ddd, *J* = 4.9, 1.9, 0.9 Hz, 1H), 7.89 (td, *J* = 7.7, 2.0 Hz, 1H), 7.80 (dd, *J* = 8.0, 1.5 Hz, 1H), 7.74 − 7.70 (m, 2H), 7.63 − 7.59 (m, 2H), 7.56 (ddd, *J* = 8.6, 7.1, 1.5 Hz, 1H), 7.52 − 7.48 (m, 2H), 7.37 (ddd, *J* = 8.1, 7.2, 1.2 Hz, 1H), 7.27 (ddd, *J* = 7.5, 4.8, 1.0 Hz, 1H), 5.14 (s, 2H), 2.48 (s, 3H), ^13 ^C NMR (176 MHz, DMSO-*d*_6_) δ 172.54, 165.96, 157.95, 154.81, 154.18, 149.22, 142.85, 139.16, 133.41, 132.44, 130.93, 130.19 (2 C), 129.56, 129.27, 123.94, 122.89, 122.73, 118.99 (2 C), 115.17, 45.81, 21.57; Anal. Calcd. for C_23_H_19_N_5_O_3_ (413.44): C, 66.82; H, 4.63; N, 16.94; Found: C, 66.97; H, 4.46; N, 16.79%.

##### 4.1.1.10. 4–(2-(3-Methyl-2-oxoquinoxalin-1(2H)-yl)acetamido)-N-(thiazol-2-yl)benzamide 11j

Brown crystals (yield 71%); mp: 230 − 232 °C; FT-IR (v max, cm^−1^): 3413 (NH), 1660 (C=O), 1602 (C = N); ^1^H NMR (700 MHz, DMSO-*d*_6_) δ 12.51 (s, 1H), 10.94 (s, 1H), 8.14 (d, *J* = 8.4 Hz, 1H), 8.10 (d, *J* = 8.7 Hz, 1H), 7.82 − 7.75 (m, 2H), 7.73 (d, *J* = 8.5 Hz, 1H), 7.61 − 7.55 (m, 3H), 7.29 − 7.25 (m, 1H), 7.06 − 7.04 (m, 1H), 5.19 (d, *J* = 6.4 Hz, 2H), 2.49 (s, 3H); ^13 ^C NMR (176 MHz, DMSO-*d*_6_) δ 172.05, 167.52, 166.18, 165.96, 157.97, 142.04, 133.45, 132.14, 130.34, 130.20, 129.85(2 C), 129.82, 129.04, 118.90, 118.60(2 C), 115.20, 114.21, 108.50, 45.84; MS (*m/z*): 420 (M^+^ + 1, 100%); Anal. Calcd. for C_21_H_17_N_5_O_3_S (419.46): C, 60.13; H, 4.09; N, 16.70; Found: C, 60.55; H, 3.68; N, 16.32%.

#### 4.1.2. General procedure for synthesis of compounds 12a-k

To a solution of the potassium salt of 3-methylquinoxaline-2-thiol **4** (352 mg, 0.002 mol) in DMF (20 ml) the appropriate 4–(2-chloroacetamido)-*N*-substituted-benzamides **10a-k** (0.002 mol) was added. The mixture was heated on a water bath for 6 h. After cooling to room temperature, the reaction mixture was poured onto crushed ice. The precipitated solids were filtered, dried, and crystalised from ethanol to give the target compounds **12a-k.**

##### 4.1.2.1. N-Methyl-4–(2-((3-methylquinoxalin-2-yl)thio)acetamido)benzamide 12a

Brown powder (yield 65%); mp: 219 − 221 °C; FT-IR (v max, cm^−1^): 3298 (NH), 2923 (CH aliphatic), 1649 (C=O), 1604 (C = N); ^1^H NMR (700 MHz, DMSO-*d*_6_) δ 10.71 (s, 1H), 8.38 (q, *J* = 4.6 Hz, 1H), 7.85 − 7.80 (m, 3H), 7.68 (d, *J* = 8.4 Hz, 2H), 7.59 (t, *J* = 8.4 Hz, 1H), 7.55 (d, *J* = 8.4 Hz, 1H), 7.40 (t, *J* = 7.5 Hz, 1H), 5.19 (s, 2H), 2.79 (s, 3H), 2.30 (s, 3H); ^13 ^C NMR (176 MHz, DMSO) δ 166.48, 165.70, 157.99, 154.86, 141.48, 133.48, 132.47, 130.19, 129.89, 129.28, 128.48 (2 C), 123.95, 118.85(2 C), 115.20, 45.76, 26.69, 21.61; Anal. Calcd. for C_19_H_18_N_4_O_2_S (366.44): C, 62.28; H, 4.95; N, 15.29; Found: C, 62.53; H, 5.42; N, 15.88%.

##### 4.1.2.2. N-(sec-Butyl)-4–(2-((3-methylquinoxalin-2-yl)thio)acetamido)benzamide 12b

Brown powder (yield 65%); mp: 165–167 °C; FT-IR (v max, cm^−1^): 3286 (NH), 2965 (CH aliphatic), 1631 (C=O), 1608 (C = N); ^1^H NMR (700 MHz, DMSO-*d*_6_) δ 10.66 (s, 1H), 8.05 (d, *J* = 8.2 Hz, 1H), 8.02 (d, *J* = 8.2 Hz, 1H), 7.96 (dd, *J* = 8.1, 1.5 Hz, 1H), 7.83 (d, *J* = 8.5 Hz, 2H), 7.82 (s, 1H), 7.72 (d, *J* = 6.9 Hz, 1H), 7.69 (d, *J* = 8.7 Hz, 2H), 4.30 (s, 2H), 3.91 (m, *J* = 7.1 Hz, 2H), 2.67 (s, 3H), 1.51 (dt, *J* = 22.9, 7.2 Hz, 2H), 1.13 (d, *J* = 6.6 Hz, 3H), 0.86 (t, *J* = 7.4 Hz, 3H); ^13 ^C NMR (176 MHz, DMSO-*d*_6_) δ 166.91, 165.53, 155.45, 151.96, 141.86, 140.81, 139.35, 130.09, 130.02, 128.89, 128.67, 128.62(2 C),127.37, 118.65(2 C), 46.79, 35.38, 29.34, 22.18, 20.78, 11.24; MS (*m/z*): 409 (M^+^ + 1, 100%, base beak); Anal. Calcd. for C_22_H_24_N_4_O_2_S (408.52): C, 64.68; H, 5.92; N, 13.71; Found: C, 64.99; H, 5.77; N, 13.50%.

##### 4.1.2.3. N-Cyclopentyl-4–(2-((3-methylquinoxalin-2-yl)thio)acetamido)benzamide 12c

Grey powder (yield 72%); mp: 220 − 222 °C; FT-IR (v max, cm^−1^): 3285 (NH), 2954, 2866 (CH aliphatic), 1668, 1629 (C=O), 1607 (C = N); ^1^H NMR (700 MHz, DMSO-*d*_6_) δ 10.66 (s, 1H), 8.15 (d, *J* = 7.2 Hz, 1H), 7.96 (dd, *J* = 8.1, 1.6 Hz, 1H), 7.85 − 7.80 (m, 3H), 7.71 (ddd, *J* = 8.3, 6.9, 1.6 Hz, 1H), 7.68 (dq, *J* = 9.8, 3.0, 2.2 Hz, 3H), 4.30 (s, 2H), 4.25 − 4.18 (m, 1H), 2.67 (s, 3H), 1.91 − 1.84 (m, 2H), 1.74 − 1.65 (m, 2H), 1.57 − 1.48 (m, 4H); ^13 ^C NMR (176 MHz, DMSO-*d*_6_) δ 166.91, 165.79, 155.45, 151.97, 141.86, 140.81, 139.35, 130.02, 129.97, 128.90, 128.69, 128.67(2 C), 127.37, 118.61(2 C), 51.33, 35.38, 32.61(2 C), 24.09(2 C), 22.18; MS (*m/z*): 421 (M^+^ + 1, 100%); Anal. Calcd. for C_23_H_24_N_4_O_2_S (420.53): C, 65.69; H, 5.75; N, 13.32; Found: C, 65.23; H, 5.50; N, 13.02%.

##### 4.1.2.4. N-(2-Methoxyphenyl)-4–(2-((3-methylquinoxalin-2-yl)thio)acetamido) benzamide 12d

Buff powder (yield 72%); mp: 185–187 °C; FT-IR (v max, cm^−1^): 3420 (NH), 1698, 1645 (C=O), 1602 (C = N); ^1^H NMR (700 MHz, DMSO-*d*_6_) δ 10.75 (s, 1H), 9.31 (s, 1H), 7.99 − 7.93 (m, 3H), 7.85 − 7.82 (m, 1H), 7.80 (dd, *J* = 7.8, 1.6 Hz, 1H), 7.78 − 7.75 (m, 2H), 7.71 (dddd, *J* = 25.1, 8.4, 7.0, 1.5 Hz, 2H), 7.18 (ddd, *J* = 8.2, 7.4, 1.7 Hz, 1H), 7.10 (dd, *J* = 8.3, 1.3 Hz, 1H), 6.97 (td, *J* = 7.6, 1.4 Hz, 1H), 4.33 (s, 2H), 3.84 (s, 3H), 2.68 (s, 3H); ^13 ^C NMR (176 MHz, DMSO-*d*_6_) δ 167.07, 164.77, 155.46, 151.98, 151.77, 142.51, 140.82, 139.36, 130.06, 129.49, 128.99, 128.92(2 C), 128.69, 127.41, 127.38, 125.97, 124.53, 120.67, 118.88(2 C), 111.80, 56.19, 35.44, 22.19; MS (*m/z*): 459 (M^+^ + 1, 70% %), 217 (100%); Anal. Calcd. for C_25_H_22_N_4_O_3_S (458.54): C, 65.49; H, 4.84; N, 12.22; Found: C, 65.11; H, 4.62; N, 12.56%.

##### 4.1.2.5. N-(3-Methoxyphenyl)-4–(2-((3-methylquinoxalin-2-yl)thio)acetamido) benzamide 12e

Reddish crystals (yield 70%); mp: 232 − 234 °C; FT-IR (v max, cm^−1^): 3277 (NH), 3039 (CH aromatic), 2989, 2926, 2827 (CH aliphatic), 1678, 1650 (C=O); ^1^H NMR (700 MHz, DMSO-*d*_6_) δ 10.76 (s, 1H), 10.11 (s, 1H), 7.96 (d, *J* = 8.7 Hz, 3H), 7.83 (dd, *J* = 8.2, 1.5 Hz, 1H), 7.80 − 7.76 (m, 2H), 7.70 (dddd, *J* = 25.4, 8.4, 6.9, 1.5 Hz, 2H), 7.48 (t, *J* = 2.3 Hz, 1H), 7.40 − 7.35 (m, 1H), 7.25 (t, *J* = 8.1 Hz, 1H), 6.70 − 6.65 (m, 1H), 4.33 (s, 2H), 3.76 (s, 3H), 2.67 (s, 3H); ^13 ^C NMR (176 MHz, DMSO-*d*_6_) δ 167.08, 165.36, 159.88, 155.43, 151.96, 142.49, 140.94, 140.82, 139.36, 130.02, 129.90, 129.81, 129.19 (2 C), 128.89, 128.68, 127.37, 118.78 (2 C), 112.97, 109.46, 106.44, 55.45, 35.44, 22.18; MS (*m/z*): 459 (M^+^ + 1, 100%); Anal. Calcd. for C_25_H_22_N_4_O_3_S (458.54): C, 65.49; H, 4.84; N, 12.22; Found: C, 65.83; H, 4.57; N, 11.94%.

##### 4.1.2.6. N-(4-Methoxyphenyl)-4–(2-((3-methylquinoxalin-2-yl)thio)acetamido) benzamide 12f

White powder (yield 71%); mp: 250–252 °C; FT-IR (v max, cm^−1^): 3300 (NH), 3054 (CH aromatic), 2909, 2834 (CH aliphatic), 1677, 1644 (C=O), 1600 (C = N); ^1^H NMR (700 MHz, DMSO-*d*_6_) δ 10.74 (s, 1H), 10.02 (s, 1H), 7.96 (t, *J* = 9.1 Hz, 3H), 7.83 (dd, *J* = 8.0, 2.8 Hz, 1H), 7.76 (dd, *J* = 8.6, 2.9 Hz, 2H), 7.72 (t, *J* = 7.5 Hz, 1H), 7.67 (dd, *J* = 9.1, 3.1 Hz, 3H), 6.93 (dd, *J* = 9.2, 3.0 Hz, 2H), 4.33 (s, 2H), 3.75 (s, 3H), 2.67 (d, *J* = 2.9 Hz, 3H); ^13 ^C NMR (176 MHz, DMSO-*d*_6_) δ 167.04, 164.91, 155.91, 155.45, 151.97, 142.30, 140.82, 139.36, 132.79, 130.03 (2 C), 129.05 (2 C), 128.89, 128.68, 127.37, 122.40 (2 C), 118.77 (2 C), 114.18 (2 C), 55.63, 35.43, 22.18; MS (*m/z*): 459 (M^+^ + 1, 100%); Anal. Calcd. for C_25_H_22_N_4_O_3_S (458.54): C, 65.49; H, 4.84; N, 12.22; Found: C, 65.02; H, 4.57; N, 11.99%.

##### 4.1.2.7. N-(4-Acetylphenyl)-4–(2-((3-methylquinoxalin-2-yl)thio)acetamido)benzamide 12g

Brown powder (yield 77%); mp: 270 − 273 °C; FT-IR (v max, cm^−1^): 3300 (NH), 2918 (CH aliphatic), 1672, 1649 (C=O), 1590 (C = N); ^1^H NMR (700 MHz, DMSO-*d*_6_) δ 10.78 (s, 1H), 10.45 (s, 1H), 7.99 (d, *J* = 2.3 Hz, 1H), 7.98 (d, *J* = 2.4 Hz, 2H), 7.97 (d, *J* = 1.9 Hz, 2H), 7.95 (s, 1H), 7.94 (d, *J* = 2.0 Hz, 1H), 7.83 (dd, *J* = 8.1, 1.6 Hz, 1H), 7.80 (d, *J* = 2.0 Hz, 1H), 7.79 (d, *J* = 1.9 Hz, 1H), 7.72 (ddd, *J* = 8.3, 6.9, 1.6 Hz, 1H), 7.68 (ddd, *J* = 8.3, 6.9, 1.6 Hz, 1H), 4.33 (s, 2H), 2.67 (s, 3H), 2.55 (s, 3H); ^13 ^C NMR (176 MHz, DMSO-*d*_6_) δ 197.05, 167.14, 165.74, 155.43, 151.97, 144.22, 142.79, 140.81, 139.36, 132.33, 130.03, 129.76 (2 C), 129.43(2 C), 128.90, 128.69, 127.37, 119.84, 119.82(2 C), 118.81(2 C), 35.44, 26.93, 22.18; MS (*m/z*): 471 (M^+^ + 1, 100%); Anal. Calcd. for C_26_H_22_N_4_O_3_S (470.55): C, 66.37; H, 4.71; N, 11.91; Found: C, 65.94; H, 4.66; N, 11.58%.

##### 4.1.2.8. N-(4-Fluorophenyl)-4–(2-((3-methylquinoxalin-2-yl)thio)acetamido)benzamide 12h

White powder (yield 72%); mp: 250 − 252 °C; FT-IR (v max, cm^−1^): 3261 (NH), 3042 CH aromatic), 2912 (CH aliphatic), 1659, 1640 (C=O), 1608 (C = N); ^1^H NMR (700 MHz, DMSO-*d*_6_) δ 10.77 (s, 1H), 10.20 (s, 1H), 7.96 (d, *J* = 8.9 Hz, 3H), 7.83 (d, *J* = 8.1 Hz, 1H), 7.79 (t, *J* = 9.1 Hz, 4H), 7.74 − 7.65 (m, 2H), 7.19 (t, *J* = 8.7 Hz, 2H), 4.33 (s, 2H), 2.67 (s, 3H); ^13 ^C NMR (176 MHz, DMSO-*d*_6_) δ 167.08, 165.26, 155.43, 151.96, 142.52, 140.82, 139.36, 130.01, 129.72, 129.17(2 C), 128.88, 128.68, 127.37, 122.60(2 C), 122.56, 118.80(2 C), 115.67(2 C), 115.54, 35.43, 22.18; MS (*m/z*): 447 (M^+^ + 1, 70% %); Anal. Calcd. for C_24_H_19_FN_4_O_2_S (446.50): C, 64.56; H, 4.29; F, 4.25; N, 12.55; Found: C, 64.12; H, 3.83; N, 12.14%.

##### 4.1.2.9. 4–(2-((3-Methylquinoxalin-2-yl)thio)acetamido)-N-(pyridin-2-yl)benzamide 12i

Grey powder (yield 70%); mp: 215 − 217 °C; FT-IR (v max, cm^−1^): 3311 (NH), 1683 (C=O), 1593 (C = N); ^1^H NMR (700 MHz, DMSO-*d*_6_) δ 10.77 (s, 1H), 10,20 (s, 1H), 8.30 (ddd, *J* = 4.8, 1.9, 0.8 Hz, 1H), 7.96 (dd, *J* = 8.0, 1.7 Hz, 1H), 7.88 (td, *J* = 7.8, 2.0 Hz, 1H), 7.81 − 7.77 (m, 1H), 7.73 − 7.71 (m, 2H), 7.69 (td, *J* = 7.8, 1.6 Hz, 2H), 7.67 − 7.65 (m, 2H), 7.49 (dt, *J* = 8.1, 1.0 Hz, 1H), 7.26 (ddd, *J* = 7.4, 4.8, 1.0 Hz, 1H), 4.29 (s, 2H), 2.66 (s, 3H); ^13 ^C NMR (176 MHz, DMSO-*d*_6_) δ 172.58, 167.24, 155.37, 154.21, 151.94, 149.20, 143.34, 140.78, 139.35, 139.15, 130.95, 130.04, 129.28, 128.92 (2 C), 128.67, 127.37, 122.84, 122.68, 118.94 (2 C), 35.42, 22.17; MS (*m/z*): 430 (M^+^ + 1, 80%), 119 (100%); Anal. Calcd. for C_23_H_19_N_5_O_2_S (429.50): C, 64.32; H, 4.46; N, 16.31; Found: C, 64.61; H, 4.84; N, 15.92%.

##### 4.1.2.10. 4–(2-((3-Methylquinoxalin-2-yl)thio)acetamido)-N-(m-tolyl)benzamide 12j

Buff powder (yield 73%); mp: 242–244 °C; FT-IR (v max, cm^−1^): 3287 (NH), 1674, 1643 (C=O), 1591 (C = N); ^1^H NMR (700 MHz, DMSO-*d*_6_) δ 10.74 (s, 1H), 10.05 (s, 1H), 7.99 − 7.93 (m, 3H), 7.84 (dd, *J* = 8.1, 1.6 Hz, 1H), 7.79 − 7.75 (m, 2H), 7.71 (dddd, *J* = 24.1, 8.3, 7.0, 1.5 Hz, 2H), 7.61 (t, *J* = 2.0 Hz, 1H), 7.58 − 7.54 (m, 1H), 7.23 (t, *J* = 7.8 Hz, 1H), 6.94 − 6.90 (m, 1H), 4.33 (s, 2H), 2.68 (s, 3H), 2.31 (s, 3H); ^13 ^C NMR (176 MHz, DMSO-*d*_6_) δ 167.06, 165.25, 155.45, 151.98, 142.42, 140.82, 139.65, 139.36, 138.16, 130.04, 129.96, 129.16 (2 C), 128.92, 128.88, 128.69, 127.38, 124.67, 121.33, 118.77 (2 C), 117.97, 35.43, 22.19, 21.70; MS (*m/z*): 443 (M^+^ + 1, 100%); Anal. Calcd. for C_25_H_22_N_4_O_2_S (442.54): C, 67.85; H, 5.01; N, 12.66; Found: C, 67.53; H, 4.94; N, 12.38%.

##### 4.1.2.11. 4–(2-((3-Methylquinoxalin-2-yl)thio)acetamido)-N-(p-tolyl)benzamide 12k

White powder (yield 77%); mp: 259 − 261 °C; FT-IR (v max, cm^−1^): 3291, (NH), 3036 (CH aromatic), 2982, 2922 (CH aliphatic), 1661, 1641 (C=O), 1596 (C = N); ^1^H NMR (700 MHz, DMSO-*d*_6_) δ 10.74 (s, 1H), 10.05 (s, 1H), 7.96 (dd, *J* = 10.2, 8.4 Hz, 3H), 7.83 (dd, *J* = 8.1, 1.5 Hz, 1H), 7.77 (d, *J* = 8.5 Hz, 2H), 7.73 − 7.70 (m, 1H), 7.68 (ddd, *J* = 8.3, 7.0, 1.5 Hz, 1H), 7.65 (d, *J* = 8.2 Hz, 2H), 7.15 (d, *J* = 8.2 Hz, 2H), 4.33 (s, 2H), 2.67 (s, 3H), 2.28 (s, 3H); ^13 ^C NMR (176 MHz, DMSO-*d*_6_) δ 167.05, 165.13, 155.45, 151.97, 142.37, 140.82, 139.36, 137.20, 132.90, 130.03, 130.01, 129.43(2 C), 129.12 (2 C), 128.90, 128.68, 127.37, 120.81 (2 C), 118.77 (2 C), 35.43, 22.19, 20.96; MS (*m/z*): 443 (M^+^ + 1, 100%); Anal. Calcd. for C_25_H_22_N_4_O_2_S (442.54): C, 67.85; H, 5.01; N, 12.66; Found: C, 67.45; H, 4.89; N, 12.21%.

### 4.2. Biological testing

#### 4.2.1. *In vitro* cytotoxic activity

*In vitro* cytotoxicity was carried out using MTT assay protocol^47^^,^^65–67^ as described in Supplementary data.

#### 4.2.2. *In vitro* VEGFR-2 kinase assay

*In vitro* VEGFR-2 inhibitory activity was assessed against. Human VEGFR-2 ELISA kit as described in Supplementary data^68^^,^^69^.

#### 4.2.3. *In vitro* cytotoxicity against normal cell

The toxicity of compounds **11e** and **12e** was assessed against normal cell lines (primary rat hepatocytes) according to method of two-steps *in situ* collagenase perfusion as described by Seglen^70^ (Supplementary data).

#### 4.2.4. Cell cycle analysis

The effect of compound **11e** on cell cycle distribution was performed using propidium iodide (PI) staining technique as described in Supplementary data^71–73^.

#### 4.2.5. Apoptosis analysis

The effect of compound **11e** on cell apoptosis was investigated as described in Supplementary data^74–76^.

#### 4.2.6. Western blot analysis

Western blot technique was applied to assess the potential effect of compound **11e** on the expression of caspase‐9, caspase‐3, BAX, and Bcl‐2 as reported in Supplementary data^77–79^.

### 4.3. *In silico* studies

#### 4.3.1. Docking studies

Crystal structure of VEGFR-2 [PDB ID: PDB ID: 2OH4, resolution: 2.05 Å] was obtained from Protein Data Bank. The docking investigation was accomplished using MOE2014 software. At first, the crystal structure of VEGFR-2 was prepared by removing water molecules. Only one chain was retained beside the co-crystallized ligand (sorafenib). Then, the selected chain was protonated and subjected to minimisation of energy process. Next, the active site of the target protein was defined.

Structures of the synthesised compounds and sorafenib were drawn using ChemBioDraw Ultra 14.0 and saved as MDL-SD format. Such file was opened using MOE to display the 3 D structures which were protonated and subjected to energy minimisation. Formerly, validation of the docking process was performed by docking the co-crystallized ligand against the isolated pocket of active site. The produced RMSD value indicated the validity of process. Finally, docking of the tested compounds was done through the dock option inserted in compute window. For each docked molecule, 30 docked poses were produced using ASE for scoring function and force field for refinement. The results of the docking process were then visualised using Discovery Studio 4.0 software^34^^,^^80–83^.

#### 4.3.2. ADMET studies

ADMET descriptors were determined using Discovery studio 4.0 as according the reported method^34^^,^^80^^,^^84^ (Supplementary data).

#### 4.3.3. Toxicity studies

Discovery studio 4.0 software was used to predict the toxicity potential of the synthesised compounds as reported in Supplementary data^85^.
